# Luminescent 1,10-Phenanthroline β-Diketonate Europium Complexes with Large Second-Order Nonlinear Optical Properties

**DOI:** 10.3390/molecules27206990

**Published:** 2022-10-18

**Authors:** Francesco Fagnani, Alessia Colombo, Graziella Malandrino, Claudia Dragonetti, Anna Lucia Pellegrino

**Affiliations:** 1 Dipartimento di Chimica, Università di Milano and INSTM UdR Milano, 20126 Milan, Italy; 2 Dipartimento di Scienze Chimiche, Università di Catania and INSTM UdR Catania, 95125 Catania, Italy

**Keywords:** europium complexes, antenna-ligand, second-order nonlinear optics

## Abstract

Substitution of the diglyme ligand of [Eu(hfa)_3_(diglyme)] (where hfa is hexafluoroacetylacetonate) with a simple 1,10-phenanthroline leads to a six-fold increase of the product μβ_EFISH_, as measured by the Electric-Field-Induced Second Harmonic generation (EFISH) technique. Similarly, [Eu(tta)_3_(1,10-phenanthroline)] (where Htta is 2-thenoyltrifluoroacetone) is characterized by a large second-order NLO response. Both 1,10-phenanthroline europium complexes have great potential as multifunctional materials for photonics.

## 1. Introduction

The design and characterization of molecules with a second-order nonlinear optical (NLO) response have received considerable attention due to their application in a range of fields such as optical communications, electrooptical devices, and optical data processing [1,2]. To present good second-order NLO properties, a molecule has to be non-centrosymmetric, with a great difference between the excited state and the ground state dipole moment; in addition, it has to be characterized by charge-transfer transitions at relatively low energy. Thus, organic molecules containing electron-acceptor and electron-donor groups connected by a polarizable π-conjugated bridge can reach a good NLO response [1,2,3,4,5,6]. In the last twenty years, coordination compounds, characterized by low-energy ligand-to-metal, metal-to-ligand, ligand-to-ligand and intraligand charge-transfer (LMCT, MLCT, LLCT, and ILCT, respectively) excited states, have shown their great potential for second-order nonlinear optics [7,8,9,10,11,12,13,14,15,16,17,18,19,20,21,22]. In the design of NLO-active complexes, a useful aspect is that the energy of the charge-transfer states is easily controlled by the nature and oxidation state of the metal and by the choice of the ligands. In particular, it appeared that the second-order NLO response of various π-delocalized nitrogen donor ligands such as pyridines, stilbazoles, phenanthrolines, bipyridines, and terpyridines can be greatly enhanced upon coordination to a metal center [8,9,10,11,12,13,14,15,20,22,23,24,25,26]. Coordination complexes with various metals have been studied. However, surprisingly, although lanthanide (Ln) complexes have been intensively investigated for their luminescent and magnetic properties [27,28], as well as for biological applications [29,30,31,32,33], there are only a few reports on their peculiar NLO properties [34,35,36,37,38,39,40,41,42,43].

It was reported that dipolar lanthanide complexes such as [LLn(NO_3_)_3_] (where L is a dibutylaminophenyl-functionalized annelated terpyridine) are characterized by a good second-order NLO response, measured by the Harmonic Light Scattering (HLS) technique in solution [44,45,46,47], which increases as the number of f electrons increases [34,36]. Similarly, the increase in the quadratic hyperpolarizability, β_HLS_, of Na_3_[Ln(pyridyl-2,6-dicarboxylate)_3_] along the Ln series can be explained by the increased number of f electrons [35]. The unexpected fact that the quadratic hyperpolarizability depends on the number of f electrons was attributed to the polarization of the 4f electrons [41]. Additionally, it has been reported that the quadratic hyperpolarizibilities of Ln complexes bearing nonadentate ligands based on triazacyclononane, functionalized with pyridyl-2-phosphinate groups, reach a maximum around the center of the lanthanide series, with a bell-shaped trend [42]. A similar trend of the quadratic hyperpolarizibilities was observed in the case of Ln complexes of *trans*-cinnamic acid [44]. In parallel, some of us studied the second-order nonlinear optical response of [Ln(hfa)_3_(diglyme)] (hfa = hexafluoroacetylacetonate; diglyme = bis (2-methoxyethyl) ether) by a combination of Electric-Field Induced Second Harmonic generation (EFISH) and HLS techniques in solution, confirming the role of f electrons in controlling the second-order NLO properties [38]. In these systems, the molecular quadratic hyperpolarizabilities measured by the EFISH method [48], β_EFISH_, initially increase rapidly with the number of f electrons, whereas the increase is much lower for the last seven f electrons; additionally, the β_HLS_ values increase, but much less rapidly, along the Ln series [38]. Similarly, the β_EFISH_ values of trinuclear lanthanide adducts [Ln(NO_3_)_3_(CuL)_2_] (Ln = La, Ce, Sm, Eu, and Er; L = N,N′-1,3-propylen-bis (salicylideniminato)) are significantly influenced by the number of f electrons: the values initially increase rapidly with the number of f electrons, starting from lanthanum to europium; then the increase is less marked upon addition of the other f electrons, with the β_EFISH_ value of the Er complex (11 f electrons) being only 1.1 times higher than that of the Eu complex (6 f electrons) [39]. This study confirmed that the surprising polarizable character of f electrons is the origin of the fascinating NLO properties. As general trend, the increase of the second-order NLO response is significant up to fulfilment of half f shell, while it becomes much less relevant with the addition of further f electrons up to the total fulfilment of the f shell [39].

This latter observation and the fact that lighter lanthanides (Ce-Eu) are more abundant than the heavier ones (Gd-Lu), and therefore are generally less expensive [49], render europium complexes of particular interest for NLO studies. 

In the present work, we found that two known luminescent β-diketonate europium complexes [Eu(hfa)_3_(1,10-phenanthroline)] and [Eu(tta)_3_(1,10-phenanthroline)], from now on [Eu(hfa)_3_(phen)] and [Eu(tta)_3_(phen)], show an unexpected large NLO response, much higher than that previously reported for the related [Eu(hfa)_3_(diglyme)] [38], as evidenced by the Electric-Field Induced Second Harmonic generation (EFISH) technique in solution [41], opening a promising route for easily prepared multifunctional NLO-active lanthanide complexes. 

**Figure 1:**
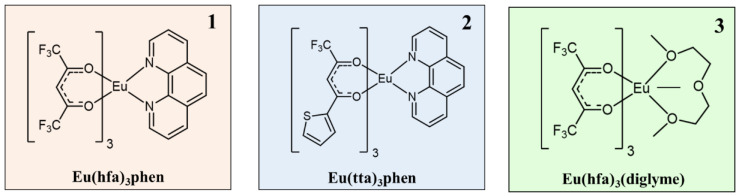
Molecular structures of (**1**) [Eu(hfa)_3_(phen)], (**2**) [Eu(tta)_3_(phen)] and (**3**) [Eu(hfa)_3_(diglyme)] complexes.

The Eu complexes under investigation are schematized in Figure 1. The structures present the Eu as central metal in the most stable oxidation state 3+ and stabilized by six oxygens coming from the β-diketonate ligands and by two nitrogens of the phenanthroline for [Eu(hfa)_3_(phen)] and [Eu(tta)_3_(phen)] (complexes **1** and **2** in Figure 1), while the Eu coordination sphere is completed with three additional oxygens of the polyether for [Eu(hfa)_3_(diglyme)], complex **3**.

## 2. Results and Discussion

The europium fluorinated β-diketonate complexes have been obtained through a facile synthesis starting from the europium acetate and the ligands. The present approach finds counterparts in the route previously reported for the analogous [Eu(hfa)_3_(diglyme)] complex [28,38] and offers several advantages, such as high yield, synthesis in a single step, low-cost route from commercially available chemicals. Additionally, all the complexes can be handled in air, are non-hygroscopic, soluble in common organic solvents and present high thermal and chemical stability.

Furthermore, the second-order nonlinear optical properties in chloroform solution of complexes **1** and **2** have been deeply studied, working with an incident radiation of low energy (λ = 1.907 µm), by the EFISH method in solution [48].

This technique, suitable for dipolar molecules, provides information on the molecular NLO properties through the following equation:

γEFISH = (μβ_λ_/5kT) + γ (*−*2ω; ω, ω, 0)

where μβ_λ_/5kT is the dipolar orientational contribution and γ (−2ω; ω, ω, 0) is the electronic cubic contribution, which can usually be neglected when studying the second-order NLO properties of dipolar molecules. Β_λ_ is the projection along the dipole moment axis of β_VEC_, which is the vectorial component of the tensor of the quadratic hyperpolarizability, working with an incident wavelength λ of a pulsed laser. To avoid overestimation of the quadratic hyperpolarizability value, due to resonance enhancements, it is necessary to work with an incident wavelength λ whose second harmonic λ/2 is far from any absorption band of the compound investigated. For this reason, a wavelength of 1.907 µm was chosen to study complexes **1** and **2**. To obtain the value of β_EFISH_ it would be necessary to know the ground state dipole moment μ of the molecule. However, from an applicative point of view, it is the product μβ_EFISH_ that should be maximized. A compound with a μβ_E__FISH_ value higher than that of Disperse Red One (500 × 10*^−^*^48^ esu), proposed for electrooptic polymeric poled films [50,51], can be considered of interest for photonic applications.

It turned out that [Eu(hfa)_3_(phen)] (**1**; Figure 1) is characterized, in solution, by a μβ_EFISH_ of 1016 × 10*^−^*^48^ esu (Table 1), 6.3 times higher than that previously reported [38] for the related complex [Eu(hfa)_3_(diglyme)] (**3**). Therefore, remarkably, replacement of diglyme with a simple 1,10-phenanthroline leads to a huge increase of the second-order NLO properties. Such a large NLO response is also observed for [Eu(tta)_3_(phen)] (**2**; μβ_EFISH_ = 920 × 10*^−^*^48^ esu) in which the hexafluoroacetylacetonate ligand has been replaced by the 2-thenoyltrifluoroacetonate. It is worth pointing out that these values are higher than that observed for the europium adduct [Eu(NO_3_)_3_(CuL)_2_] in solution (μβ_EFISH_ = 720 × 10*^−^*^48^ esu), which is much more difficult to prepare [39].

The large NLO response of **1** and **2** is thrilling also due to the simplicity of the 1,10-phenanthroline ligand. In fact, it is known that coordination of 5-X-1,10-phenanthrolines to a “Zn(CH_3_CO_2_)_2_” moiety produces a significant enhancement of the product μβ_EFISH_, which becomes 99 × 10*^−^*^48^, 254 × 10*^−^*^48^, and 616 × 10*^−^*^48^ esu for X = OMe, NMe_2_, and trans-CH=CHC_6_H_4_NMe_2_, respectively) [52,53], but the best NLO response of these Zn(II) complexes is lower than that obtained for complexes **1** and **2**, although the 1,10-phenanthroline ligand is functionalized in the Zn systems. 

**Figure 2:**
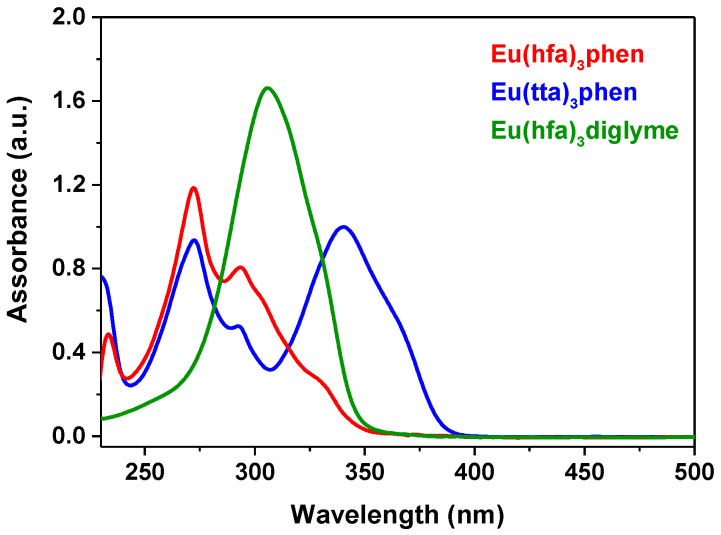
UV-vis spectra of [Eu(hfa)_3_(phen)], [Eu(tta)_3_(phen)] and [Eu(hfa)_3_(diglyme)], complexes from 1 × 10*^−^*^4^ M solutions in CH_2_Cl_2_.

The absorption spectra of the complexes, reported in Figure 2, display various features as a function of the different ligands which compose the structures. 

**Figure 3:**
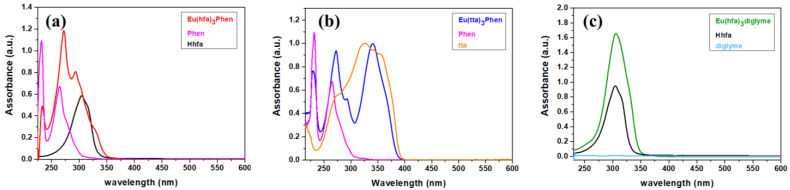
UV-vis spectra comparison of (**a**) [Eu(hfa)_3_(phen)], (**b**) [Eu(tta)_3_(phen)] and (**c**) [Eu(hfa)_3_(diglyme)] complexes with the corresponding ligands (1 × 10*^−^*^4^ M CH_2_Cl_2_).

In particular, the [Eu(hfa)_3_(diglyme)] adduct shows a strong band around 306 nm, whereas the [Eu(hfa)_3_(phen)] presents bands centered at 233, 272 and 293 nm arising from the phen and hfa contributions. Notably, in both complexes, a shoulder around 325 nm can be assigned to the lowest spin-allowed π-π* transition of the β-diketonate hfa ligand [54,55]. Finally, the absorption spectrum of the [Eu(tta)_3_(phen)] displays, together with the bands at 230 and 272 nm due to the 1,10-phenanthroline, a broad and intense signal at 341 nm arising from the tta ligand. For an easier comparison of the different contribution arising from each ligand, overlays of the UV-vis spectra of the complexes and the associated ligands are reported in Figure 3. Thus, the UV-vis spectra of **1** and **2** are due to the contribution of the relative β-diketonate, hfa and tta for **1** and **2**, respectively, and antenna ligand phen, while the UV spectrum of **3** is only due to the hfa contribution, the diglyme beeing inactive in the UV-vis region. 

**Figure 4:**
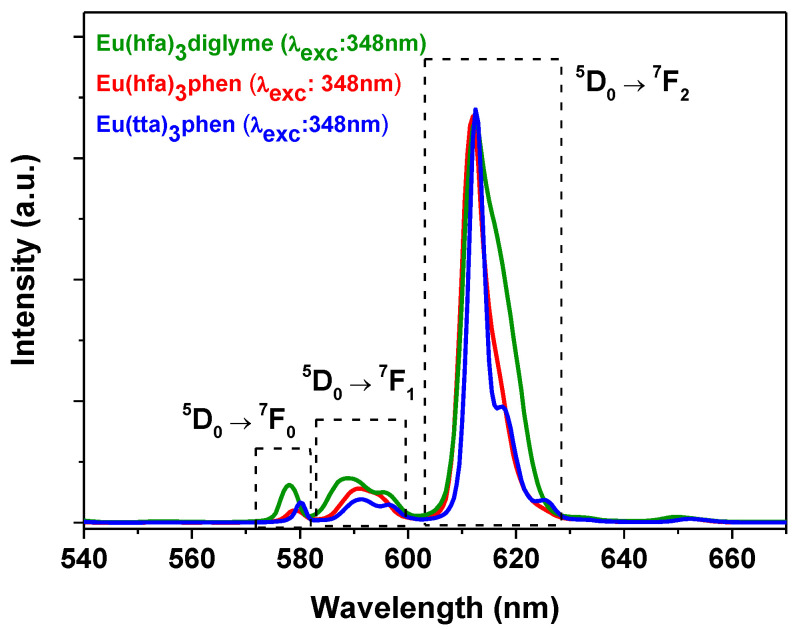
Luminescence spectra (λ_exc_ = 348 nm), of [Eu(hfa)_3_(phen)], [Eu(tta)_3_(phen)] and [Eu(hfa)_3_(diglyme)], complexes in CH_2_Cl_2_.

In addition, the luminescence spectra of the adducts, registered at room temperature, are reported in Figure 4. The spectra were obtained using an excitation wavelength of 348 nm for the [Eu(hfa)_3_(phen)], [Eu(tta)_3_(phen)] and [Eu(hfa)_3_(diglyme)].

The spectra, recorded as CH_2_Cl_2_ solutions, are reported normalized in intensity, but similar intensity values have been obtained with concentrations of 10^−3^ M, 10^−5^ M and 10^−3^ M for the complexes **1**, **2** and **3**, respectively. Therefore, the [Eu(tta)_3_(phen)] has a much higher luminescence intensity. The emission peaks observed in Figure 4 consist of f—f emission transitions from the ^5^D_0_ excited state to the ^7^F_J_ multiplet of the Eu(III) ion. In particular, the peaks at 578, 590 and 612 nm are assigned to the Eu ion transitions ^5^D_0_ → ^7^F_0_, ^5^D_0_ → ^7^F_1_ and ^5^D_0_ → ^7^F_2_, respectively [56]. The presence of the band due to the ^5^D_0_-^7^F_0_ transition in the 574–582 nm spectral region due to a singlet-to-singlet transition indicates that the Eu^3+^ ion occupies a low symmetry environment in all the three compounds [57]. This feature is also supported by the asymmetry ratio, i.e. the ratio between the ^5^D_0_ → ^7^F_2_ and ^5^D_0_ →^7^F_1_ electronic transitions, which, with a value ranging from 9.05, to 11.89 and 17.6 for **3**, **1** and **2**, respectively, indicates a highly asymmetric environment [57].

## 3. Materials and Methods

All reagents and solvents were purchased from Sigma-Aldrich and were used without further purification. 

[Eu(hfa)_3_(1,10-phenanthroline)], (**1**). The adduct was synthetized through a one-step reaction from the Eu(III) acetate hydrate, Hhfa, 1,10-phenanthroline. Specifically, 1.5797 g (4.8 mmol) of Eu(CH_3_COO)_3_∙xH_2_O was first suspended in dichloromethane (50 mL). 1,10-phenanthroline 0.8650 g (4.8 mmol) was added to the suspension. H-hfa 2.04 mL (14.4 mmol; d = 1.47 g/mL) was added under vigorous stirring after 10 min and the mixture was refluxed under stirring for 1 h. After solvent evaporation, the complex appears in form of very light orange crystals. The crystals were collected, washed several times with pentane in order to ensure a high degree of purity, and filtered. The yield was about 90%. Elemental analysis (EuC_27_H_11_O_6_N_2_F_18_): Calc: C, 34.02; H, 1.16. Found: C, 33.69; H, 1.20.

[Eu(tta)_3_(1,10-phenanthroline)], (**2**). The adduct was synthetized following a reaction similar to that of **1** from 1.5797 g (4.8 mmol) of the Eu(III) acetate hydrate suspended in ethanol (100 mL), 0.8650 g (4.8 mmol) of 1,10-phenanthroline, and 3.1993 g (14.4 mmol) of Htta. The adduct was collected after solvent evaporation, washed several times with pentane, and a yield of about 88% was obtained. Elemental analysis (EuC_36_H_20_O_6_N_2_S_3_F_9_): Calc: C, 43.43; H, 2.02. Found: C, 43.88; H, 1.97.

[Eu(hfa)_3_(diglyme)], (**3**). The adduct was synthetized following a procedure similar to that of **1**, from 1.5797 g (4.8 mmol) of the Eu(III) acetate hydrate, 2.04 mL (14.4 mmol; d = 1.47 g/mL) Hhfa and 0.69 mL (4.8 mmol; d = 0.940 g/mL) of diglyme. A similar purification process was also executed for this product. The yield was about 92%. Elemental analysis (EuC_21_H_17_O_9_F_18_): Calc: C, 27.80; H, 1.89. Found: C, 27.35; H, 1.79.

Characterization. All EFISH measurements were carried out at the Dipartimento di Chimica of the Università degli Studi di Milano, in CHCl_3_ solutions at a concentration of 1 × 10^−3^ M, working with a non-resonant incident wavelength of 1.907 µm, obtained by Raman shifting the fundamental 1.064 µm wavelength produced by a Q-switched, mode-locked Nd^3+^: YAG laser manufactured by Atalaser. The spectrometer for the EFISH measurements was a prototype made by SOPRA (France). The reported µβ_EFISH_ values are the mean of 16 successive measurements performed on the same sample. 

The UV-Vis spectra of the three adducts were collected using a JASCO V-650 UV − vis spectrophotometer, starting from 1 × 10^−4^ M solutions in CH_2_Cl_2_. Photoluminescence spectra were collected at room temperature using a JASCO FP-8300 spectrofluorimeter at a λ excitation of 348 nm for the [Eu(hfa)_3_(diglyme)], [Eu(hfa)_3_(phen)] and [Eu(tta)_3_(phen)] complexes. Elemental microanalyses were carried out using a Carlo Erba 1106 elemental analyzer.

## 4. Conclusions

Lanthanide complexes have been intensively studied for their luminescent and magnetic properties, but recently, their NLO properties have also attracted great interest. Among them, the Eu non-centrosymmetric molecules, containing the 1,10-phenanthroline antenna ligand, have been the object of interest in the present study. This work has evidenced the unexpected huge second-order NLO response of luminescent β-diketonate europium complexes bearing a simple 1,10-phenanthroline, a result of particular relevance in the search of new multifunctional building blocks for photonic nanomaterials. Their μβ_EFISH_ values, higher than that of the benchmark Disperse Red One, open the way to the use of these Eu complexes in a wide range of technological fields such as photonic applications. Thus, the 1,10-phenanthroline ligand, a well-known antenna ligand for the photoluminescence of Eu(III), plays a crucial role in boosting the NLO response of these systems. In fact, the μβ value goes from 161(×10*^−^*^48^ esu) for the [Eu(hfa)_3_(diglyme)] complex to 1061 (×10*^−^*^48^ esu) for Eu(hfa)_3_(1,10-phenanthroline), due to the substitution of diglyme by 1,10-phenanthroline. Because the two compounds have the same β-diketonate, the unique difference, and thus the factor responsible for the significant increase in the μβ value, is the phenanthroline. This observation is further supported by the μβ value of 920 (×10*^−^*^48^ esu) found for [Eu(tta)_3_(1,10-phenanthroline)]. Other important advantages of the present work are, on the one hand, the facile, one-pot, low-cost synthetic approach, and on the other hand, the non-hygroscopic, high-solubility and air stability features of the complexes, which represent added values and open a promising route for easily preparing multifunctional NLO-active lanthanide complexes. 

Finally, the present results open the door to other intriguing EFISH investigations such as the study of the effect of donor substituents on the 1,10-phenanthroline coordinated to lanthanides.

## Figures and Tables

**Table 1:** Main absorption bands in the UV-visible spectra and second-order NLO response.

Compound	Absorption ^a^ λ_max_/nm (ε/M^−1^ cm^−^^1^)	μβ (×10^−48^ esu) ^b^
**1** * 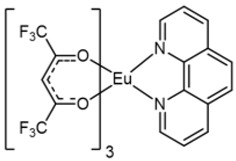 *	233, 272 (11,851), 293	1016
**2** * 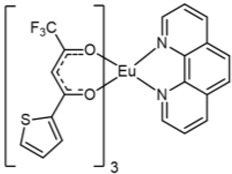 *	230, 272 (9347), 341 (10,000)	920
**3** * 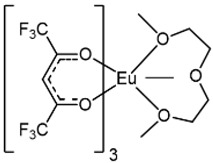 *	306 (16,617)	161

^a^ In CH_2_Cl_2_ 10*^−^*^4^ M; ^b^ In CHCl_3_ 10*^−^*^3^ M working at 1.907 μm; the experimental error is ±10%.

## Data Availability

Data is contained within the article.

## Sample Availability

Samples of the compounds are not available.
